# Analysis of the Sperm Head Protein Profiles in Fertile Men: Consistency across Time in the Levels of Expression of Heat Shock Proteins and Peroxiredoxins

**DOI:** 10.1371/journal.pone.0077471

**Published:** 2013-10-30

**Authors:** Elsa Kichine, Marcos Di Falco, Barbara F. Hales, Bernard Robaire, Peter Chan

**Affiliations:** 1 Department of Pharmacology and Therapeutics, McGill University, Montreal, Quebec, Canada; 2 Structural and Functional Genomics Centre, Concordia University, Montreal, Quebec, Canada; 3 Department of Obstetrics and Gynecology, Montreal, Quebec, Canada; 4 Department of Urology, McGill University Health Centre, Montreal, Quebec, Canada; University of Nevada School of Medicine, United States of America

## Abstract

We investigated the identity and quantitative variations of proteins extracted from human sperm heads using a label-free Gel-MS approach. Sperm samples were obtained from three men with high sperm counts at three different time points. This design allowed us to analyse intra-individual and inter-individual variations of the human sperm head proteome. Each time point was analyzed in triplicate to minimize any background artifactual effects of the methodology on the variation analyses. Intra-individual analysis using the spectral counting method revealed that the expression levels of 90% of the common proteins identified in three samples collected at various time-points, separated by several months, had a coefficient of variation of less than 0.5 for each man. Across individuals, the expression level of more than 80% of the proteins had a CV under 0.7. Interestingly, 83 common proteins were found within the core proteome as defined by the intra- and inter-variation analyses set criteria (CV<0.7). Some of these uniformly expressed proteins were chaperones, peroxiredoxins, isomerases, and cytoskeletal proteins. Although there is a significant level of inter-individual variation in the protein profiles of human sperm heads even in a well-defined group of men with high sperm counts, the consistent expression levels of a wide range of proteins points to their essential role during spermatogenesis.

## Introduction

The delivery of a genetically intact sperm nucleus during fertilization is required for normal embryo development. Subtle alterations are sufficient to disrupt the contribution of sperm DNA to the embryo [1]. The intricacy of sperm DNA packaging in mature spermatozoa results in chromatin that is distinct from that of somatic cells with a higher order of DNA compaction. The resulting condensed and tightly packaged nature of sperm chromatin protects the genetic integrity of the paternal genome during its transport through the male and female reproductive tracts [2], [3]. The condensed sperm DNA, organized in “DNA loop domains” is closely associated with the proteinaceous sperm nuclear matrix (NM) at specific sites through matrix attachment regions [4]. Although there is still little known about the functions of the NM or its protein components, there is a growing interest in studying the composition of the sperm NM as some nuclear proteins have been shown to have a role in normal sperm function [1], [5]–[9]. NM proteins are involved in paternal DNA replication in the one cell embryo [6], [7]. Ocampo et al. have shown that actin, myosin and cytokeratin are components of the NM and may ensure nuclear stability in pig spermatozoa [10]. Recently, the nuclear isoform of GPX4, nGPX4, has been implicated in matrix instability and in paternal DNA decondensation [11]. Taken together, these data strongly advocate for the importance of identifying the structural components of the NM and defining the functional roles of sperm nuclear proteins.

The use of label-free LC-MS/MS approaches is widely accepted for performing large scale quantitative analysis of proteins as a consequence of improvements in instrumentation and the development of bioinformatics tools. Two such approaches, spectral counting and ion profiling, are available. Relative quantitation by spectral counting makes use of the strong correlation between protein abundance and the number of MS/MS spectra [12]. Relative quantitation by ion profiling relies on comparison of the recorded full MS scan intensity of an eluting peptide ion at a particular chromatographic time point; it has a linear response over at least four orders of magnitude [13]. For these studies, we used both quantitation methods as a means of cross validating the results; although there are caveats associated with each of these two methodologies [14], our goal was to determine whether these methods yield similar overall results in terms of quantifiable proteins and their relative calculated ratios.

The objectives of this study were to identify the proteins in the head of human spermatozoa using a Gel-MS label-free quantitative proteomics approach, to estimate variation in protein abundance across different collection time points in three healthy normospermic men with high sperm counts, and to identify the variation and consistency of common proteins across time and among individuals.

## Materials and Methods

### Subjects and Sample Collection

Three adult men were recruited through advertisement to participate in the study.

The study was approved by the Internal Review Board of the McGill University and written informed consent was obtained from all subjects. All subjects were interviewed and examined every two to six months for a total of three visits when they provided a fresh semen sample after an abstinence period of 3–4 days. The semen samples were analyzed according to WHO protocol [15] and stored at −80 C. Three other subjects were added for the western blot analysis. Semen parameters are illustrated in Table S1 in File S1.

### Sperm Head Enrichment and Protein Extraction

Thawed frozen semen samples were washed and dissolved in 0.05 mM PBS pH 7.4 followed by three periods of sonication (40 MHz for 30 sec) in order to break off sperm tails into small pieces. Less than 15% of the sperm heads had the mid-piece attached. After centrifugation at 9,300 g for 5 min and further washing with PBS, the pellet was resuspended, first in 150 µl hypotonic buffer (Nuclear Extract Kit, Active Motif, Cedarlane Labs Ltd., Burlington, ON) and subsequently in 90 µl lysis buffer (Nuclear Extract Kit) and 10 µl of 10 mM DTT. Detergent (25 µl, Nuclear Extract Kit) was added after 15 min of incubation on ice. Proteins from the tail pieces and from potential contaminant cells were solubilised by these two incubation steps and discarded in the supernatant. After washing in PBS, the sperm head suspension was treated with Proteinase Inhibitor Cocktail (Nuclear Extract Kit) and then with 6 M guanidine, 575 mM DTT, 2 M Urea/7 M thiourea. The solubilised proteins were precipitated overnight in cold acetone and resuspended in non-complete Laemmli buffer [16] (without Bromophenol blue and 2-mercaptoethanol) and protein quantification was done in duplicate with the BioRad DC-quantification kit (BioRad, Hercules, CA). Bromophenol blue at 0.01% and ß-mercaptoethanol at 5% (final concentrations) were added and the samples were stored at −80°.

### 1D SDS-PAGE and Automated Band Excision and Digestion

Forty five micrograms of protein were separated on 2.4 cm 7 to 15% acrylamide 1D SDS-PAGE gels. The full lane was excised with a Protein Picking Workstation ProXCISION (Perkin Elmer, Waltham, MA). Thirteen gel sections were excised. Gel pieces were robotically destained, reduced, cysteine-alkylated and in-gel digested with sequencing grade modified trypsin (Promega, Madison, WI), as previously described [17]. Peptide extraction from gel section was done robotically to generate a volume of 60 µl of peptide extract per gel section.

### LC-MS/MS Analysis and Bioinformatics Data Processing

Aliquots of peptide extracts from the 13 gel sections were pooled to generate 3 pooled-sections samples. Each pooled sample was analyzed by on-line nano-HPLC-MSMS using a Velos LTQ-Orbitrap (Thermo Fisher Scientific). Peak-lists were generated using Mascot Distiller version 2.3.2.0 then searched against a database of *Homo sapiens* sequences extracted from the Universal Protein Resource (UniProt) database (September 17, 2010) containing 88352 entries, using Mascot version 2.3.01 followed by X! Tandem version 2007.01.01.1 on the subset of identified proteins. Mascot and X! Tandem searches were done using a fragment ion mass tolerance of 0.80 Da and a parent ion tolerance of 10.0 ppm. Scaffold (version 3_00_07, Proteome Software Inc., Portland, OR) was used to validate MS/MS based peptide and protein identifications. Peptide identifications were accepted if they could be established at >95.0% probability as specified by the Peptide Prophet algorithm [18]. Protein identifications were accepted if they could be established at >95.0% probability and contained at least 2 identified peptides. Protein probabilities were assigned by the Protein Prophet algorithm [19]. Proteins that contained similar peptides and could not be differentiated based on MS/MS analysis alone were grouped to satisfy the principles of parsimony. Quantitation analysis based on MS precursor ion signal was done using the precursor ion detection workflow from Proteome Discoverer Quant 1.2 (Thermo Fisher).

### Western Blotting

Total protein was extracted from the heads of spermatozoa as described above. Protein concentrations were determined using the BioRad protein assay kit according to the manufacturer’s protocol (BioRad, Mississauga, ON, Canada). Samples (5 µg/lane) were resolved by SDS polyacrylamide gradient (8%, w/v) gel electrophoresis at 100 V for 1.5 h using SDS running buffer and then transferred onto PVDF membranes. Membranes were blocked with 5% nonfat milk in TBS containing 0.1% Tween-20. Proteins were detected using antibodies specific for PPIB, PRDX5 (Santa Cruz 20361, 33573) and SPESP1 (Abcam, 72672) all diluted in 3% nonfat milk in TBS-0.1% Tween-20 followed by HRP-linked secondary antibodies; Clusterin a protein consistently expressed across all of our samples with a coefficient of variation of 0.1 was used to correct for loading.

### Statistical Analyses

Analyses of the protein profile at each time point for each subject were done in triplicate and proteins detected at a level below 3.5 assigned spectra were arbitrarily defined as artifactual signals from the technical background variation and were eliminated from further analysis. Likewise, protein signals that were not consistently present in the triplicate analysis were eliminated. Normalization of quantitative values was done by dividing the values by the mean of all quantitative values in the list of proteins in each sample. Variation in the level of expression of each protein was determined by the coefficient of variation (CV) and fold change. The intra-individual CV was calculated from the mean of triplicate analyses of the protein expression levels for each time point for each subject. The inter-individual CV of each protein was calculated from its mean level of expression at all three time points for each subject. All the statistical tests and graphs were done using GraphPad Prism version 5.00 for Windows (GraphPad Software, San Diego, CA, www.graphpad.com).

## Results

### Protein Identification and Triplicate Analyses

Analysis by LC-MS/MS of the protein extraction from the sperm head enriched preparations results in the identification of a total of 686 proteins (*table S2 in File S1*). The gene ontology analysis tool from the Scaffold software is illustrated in Fig. S1.

**Figure 1:**
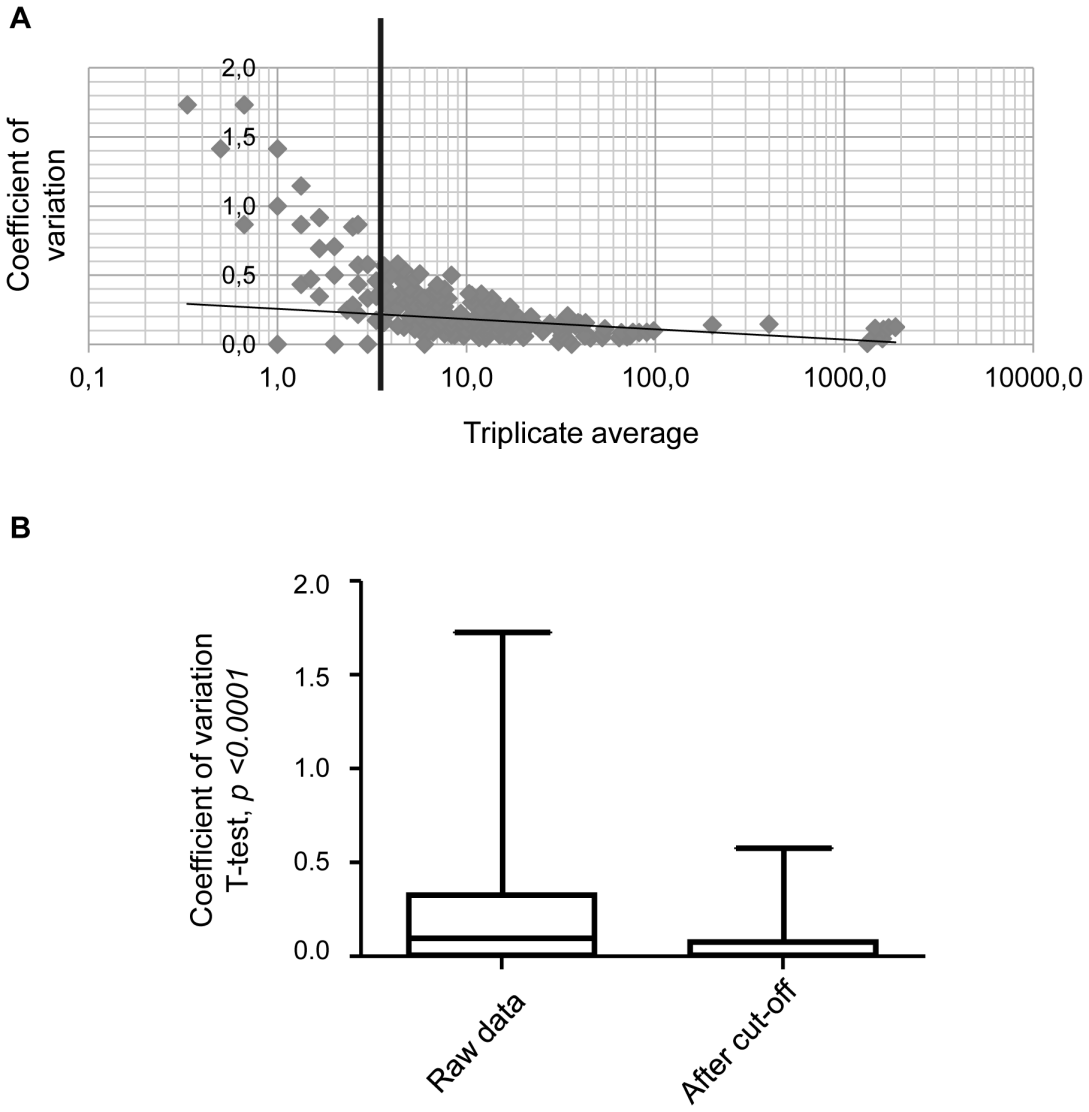
Application of the cut-off reduces the median CV of technical triplicates from 0.3 to 0.1. **A**. CV values repartition depending on the average of the triplicate quantitative value of the proteins detected in the first sample of subject 2 as an example. The highest CVs are associated with the lowest quantitative values. The cut-off has been defined at 3.5 assigned spectra. **B**. Box plot and minimum to maximum whiskers representing the decrease of the CV values after application of the cut-off.

The distribution of the CV associated with the corresponding average of the triplicates of the nine samples was analyzed. As an example, results from one of the subjects (subject 2) at the first time point are represented in Fig. 1A. The highest CVs were associated with proteins of the lowest quantitative values. Thus the variation level may be due to their higher margin of error in detection. We included in our variation analysis all proteins detected at a minimum level of 3.5 assigned spectra. As shown in Fig. 1B, the application of this cut-off significantly reduced the median CV of the total proteins analyzed from 0.3 to 0.1. This strategy ensured that the artefactual variation in proteins with low levels of detection among triplicates was minimized in the analysis.

### Intra-individual Variation in the Normal Sperm Head Proteome

**Figure 2:**
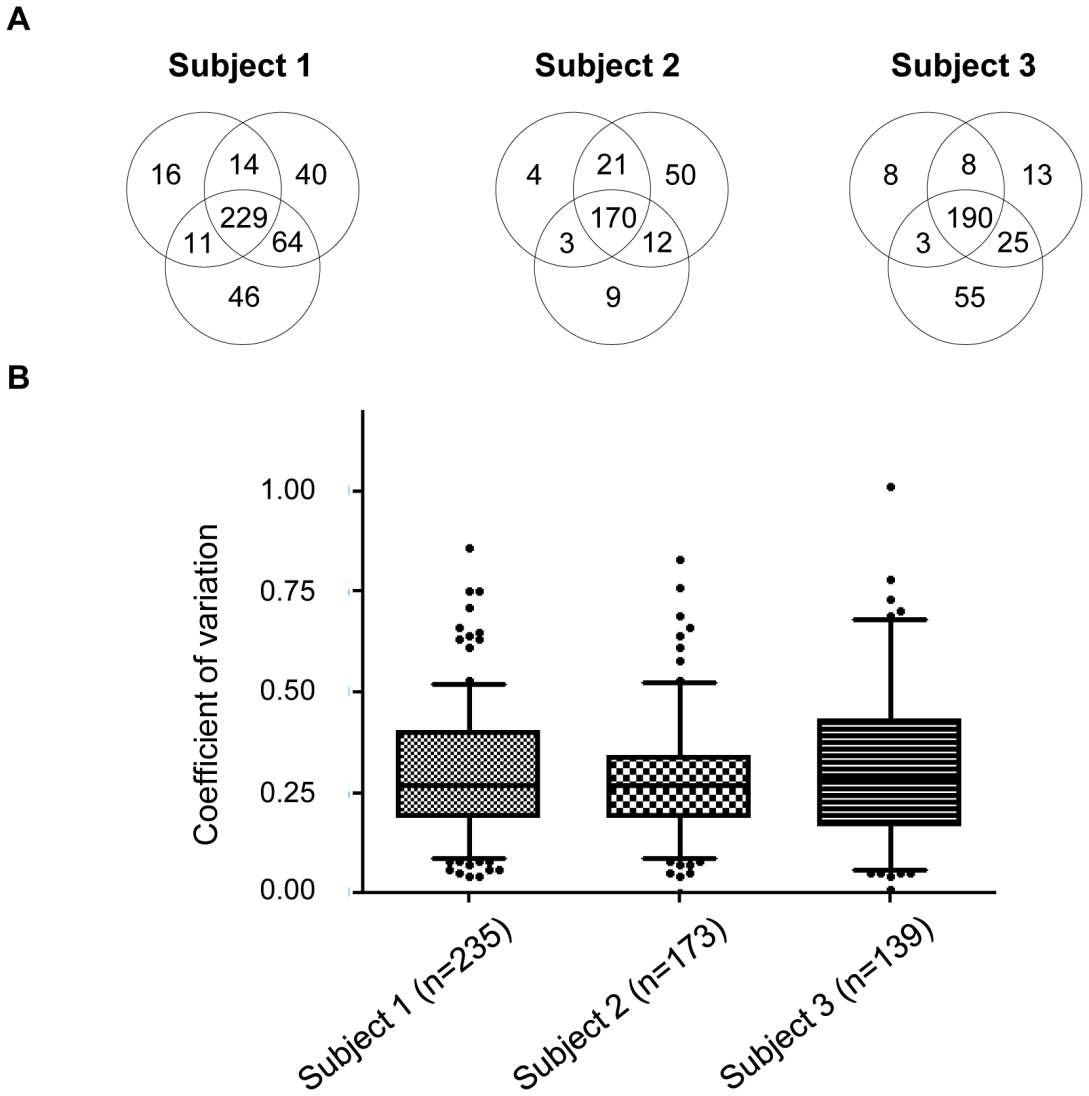
Intra-individual variation analysis. **A**. Venn diagrams of the three subjects showing the distribution of the number of identified proteins at the three time points before application of the cut-off. **B**. Box plot and 5–95 percentile whiskers illustrating the distribution of the CV values of all proteins included in the analysis of each subject. The majority of proteins had a time course CV<0.5.

The majority of proteins (55–63%) were shared across the three time points for each subject. Proteins that were found at only one time point for each subject (23%–25%) were those with the lowest total spectra abundance (Fig. 2A). Examples of these proteins were proteasome subunit (PSD7) and ribosomal proteins (RS10).

The variation analysis of proteins shared across all three time points for each subject was based on the CV values obtained from the three averages of triplicates. A boxplot with 5 to 95 percentile whiskers (Fig. 2B) was used to analyze the distribution of the CV of each protein shared across time points for each subject. An average of 90% of proteins had a lower than 0.5 dispersion level across time-points, with a median CV of 0.3 in the three subjects. Among these proteins, 33% had a low CV value (<0.2) for the time course analysis. Examples of such proteins included prolactin-inducible protein (PIP) and semenogelin 1 and 2 (SEMG1 and SEMG2) (*Table S3 in File S1*). A greater than 0.7 CV was seen in only 3% of all proteins in the analysis. These included various histones (H2AJ, H2AV, H4) and zona pellucida-binding protein 1 (ZPBP1). Interestingly, none of the various histone proteins detected were consistently associated with low or high CV values across time in the three men. Specifically, H2B1A was associated with a CV of 0.7 in subject 1, while in subject 2, the CV was only 0.3. For H2AJ the CV was 0.3 for one subject versus 0.7 in another (*Table S3 in File S1*).

These intra-individual variation analyses revealed that the majority of proteins identified were shared across all time points and that this group of proteins had a dispersion level under 0.5 across time in all subjects.

### Inter-individual Variation of the Normal Sperm Head Proteome

**Figure 3:**
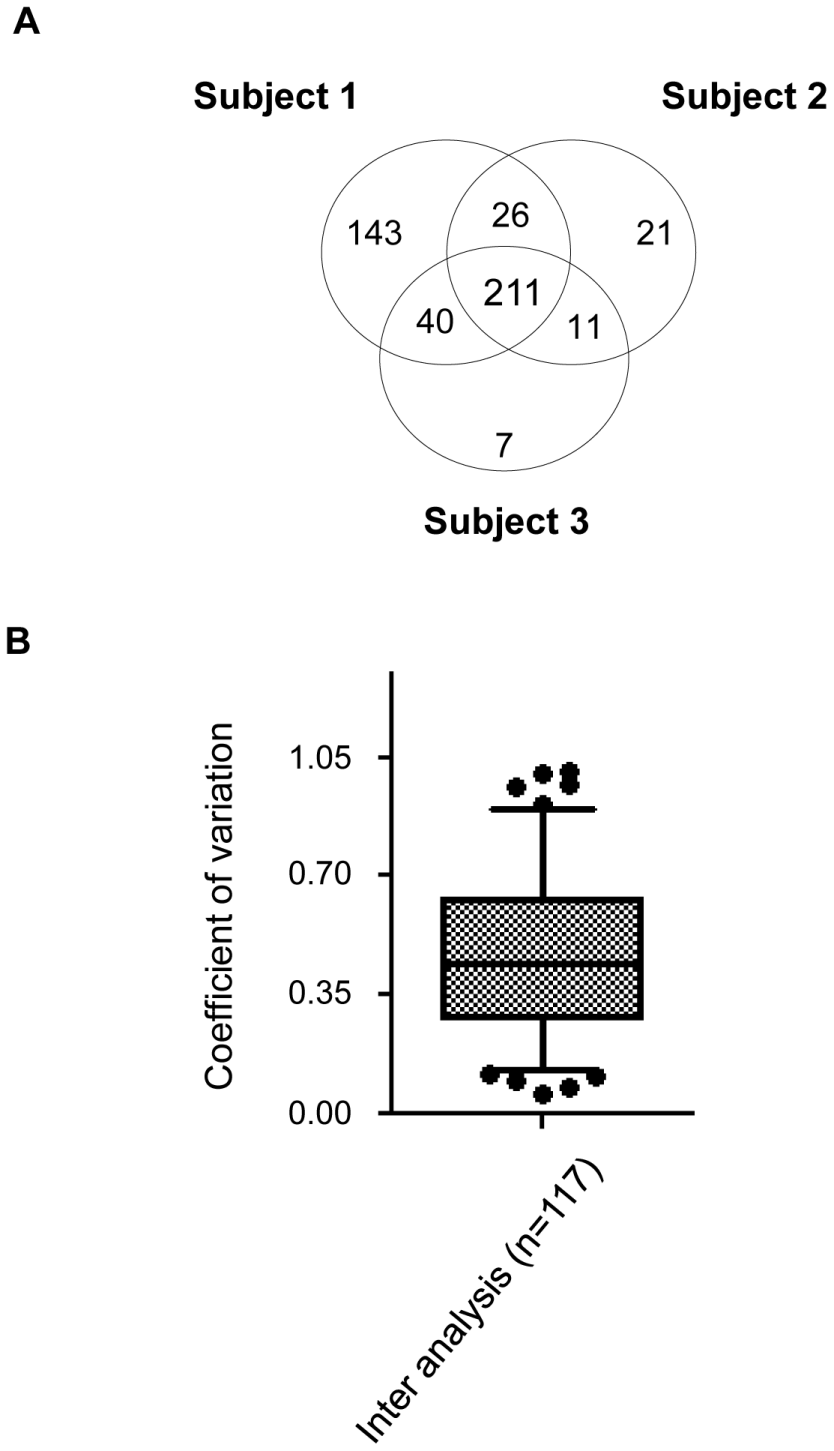
Inter-individual variation analysis. **A**. Venn diagrams of the three subjects showing the distribution of the number of identified proteins among the three men before application of the cut-off. **B**. Box plot and 5–95 percentile whiskers representing the repartition of the CV of the 117 proteins included in the analysis. The majority of these proteins had a CV<0.7.

Our inter-individual variation analysis revealed that 211 proteins were shared in all three subjects. Subject 1 had 143 unique proteins whereas subjects 2 and 3 had only 21 and 7, respectively (Fig. 3A). After applying our cut-off criteria to eliminate proteins detected at a level below 3.5 assigned spectra, a total of 117 proteins were included for further analysis. The median CV of the level of expression of these proteins was 0.5 (Fig. 3B). Among the proteins shared in all subjects, 80% had an expression level dispersion under 0.7. As expected, the expression level of the proteins shared among subjects had a higher level of dispersion than the one determined in the intra-variation analysis. Proteins such as clusterin (CLUS), SEMG1 and SEMG2, and PIP were particularly consistent in their levels of expression among men, with a CV of less than 0.2. More than 9% of the proteins were associated with a CV of more than 0.9. Some of the more variable proteins included histones H2A.J (CV = 0.8) and H2AV (CV = 0.8) and sperm equatorial segment protein 1(SPESP) (CV = 0.9) (*Table S3 in File S1).*

Therefore, inter-individual variation exceeded intra-individual variation for the proteins detected in our subjects, with 80% of proteins having dispersion in their expression level of <0.7.

### Ion profiling Quantification was Equivalent to the Spectral Count Analysis

**Figure 4:**
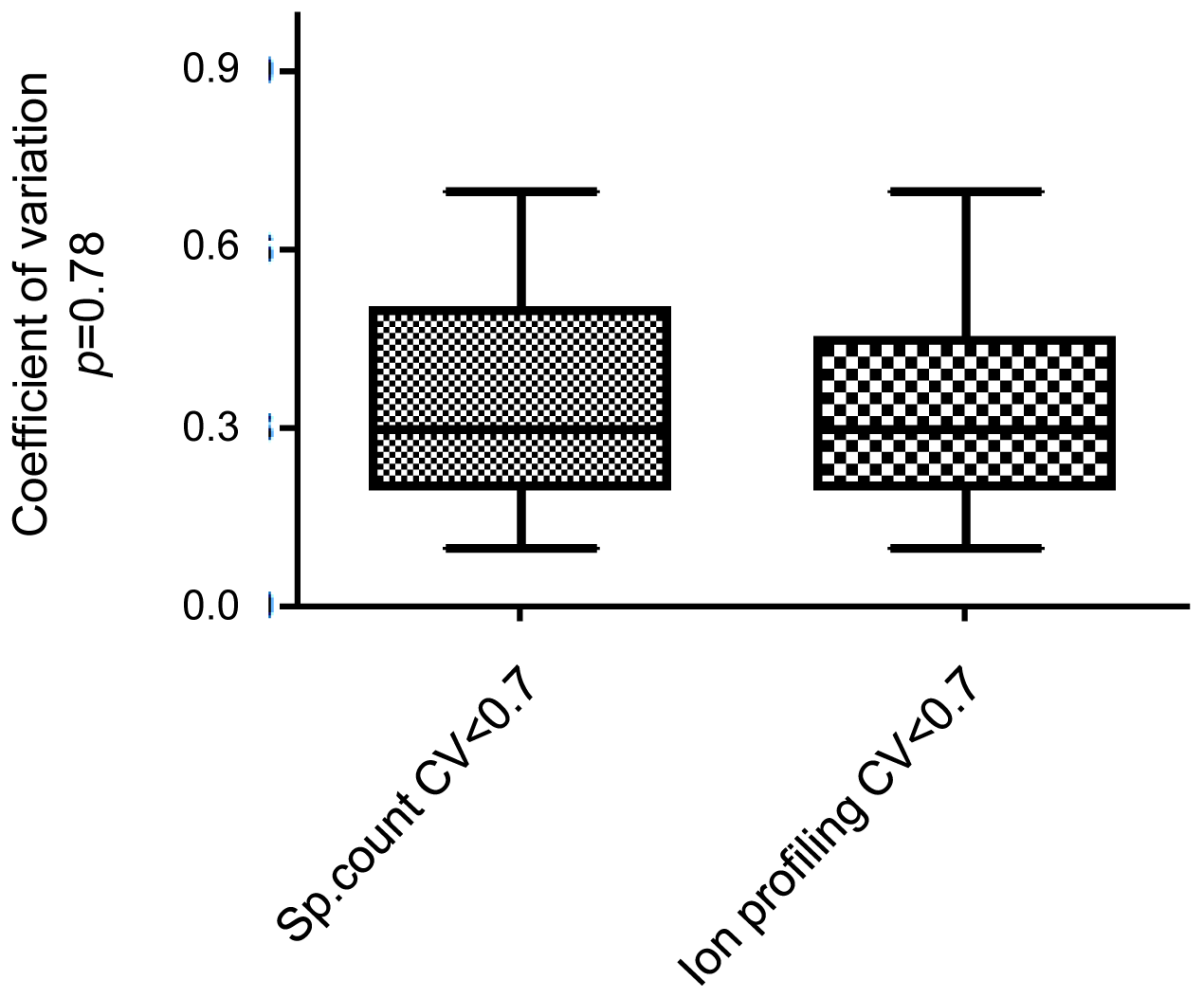
Quantification analysis using the spectral count and ion profiling methods. Box plot and minimum to maximum whiskers show repartition of the CV obtained by spectral count and ion profiling quantification methods on proteins that were determined to be commonly found in the core proteome by both intra and inter-variation analysis (CV<0.7, n = 33).

Among the 117 proteins included in the analysis, a total of 83 proteins associated with CV value of <0.7 were shared across time and among men (Table 1). These can be considered the core sperm head proteome. This consistency suggests a highly concerted regulation of their expression levels. Results of the ion profiling method of quantification were analyzed and compared to those obtained by spectral counting to further validate our observations. The CV was calculated with the same strategy described above for the inter-individual variation analyses. Of the 83 proteins listed by the spectral count quantification, 33 were also identified by ion profiling quantification and were included in the analysis. When comparing results of inter-individual analyses using spectral counting versus ion profiling quantification, we found no significant differences in the CV distribution (t-test, *p* = 0.78) (Fig. 4). These results indicate that, in our study design, the variations determined based on spectral count quantification were comparable to the values obtained by ion profiling quantification.

**Table 1:** List of 83 common proteins found within the core proteome.

Gene	Protein			Access	INTER
				Number	CV
**CLU**	**CLUS**	Clusterin		P10909	0,1
**SEMG1**	**SEMG1**	Semenogelin-1		P04279	0,1
**SEMG2**	**SEMG2**	Semenogelin-2		Q02383	0,1
**AKR1B1**	**ALDR**	Aldose reductase		P15121	0,1
**SIL1**	**SIL1**	Nucleotide exchange factor SIL1		Q9H173	0,1
**PPIB**	**PPIB**	Peptidyl-prolyl cis-trans isomerase B		P23284	0,2
**SERPINA5**	**IPSP**	Plasma serine protease inhibitor		P05154	0,2
**TUBA1A**	**TBA1A**	Tubulin alpha-1A chain		Q71U36	0,2
**AKAP4**	**AKAP4**	kinase anchor protein 4		Q5JQC9	0,2
**PRDX2**	**PRDX2**	Peroxiredoxin-2		P32119	0,2
**PRDX4**	**PRDX4**	Peroxiredoxin-4		Q13162	0,2
**ACTB**	**ACTB**	Actin, cytoplasmic 1		P60709	0,2
**CAMP**	**CAMP**	Cathelicidin antimicrobial peptide		P49913	0,2
**ANPEP**	**AMPN**	Aminopeptidase N		P15144	0,2
**TUBA3C**	**TBA3C**	Tubulin alpha-3C/D chain		Q13748	0,2
**PIP**	**PIP**	Prolactin-inducible protein		P12273	0,2
**SORD**	**DHSO**	Sorbitol dehydrogenase		Q00796	0,2
**ZG16B**	**ZG16B**	Zymogen granule protein 16 homolog B		Q96DA0	0,2
**NEU1**	**NEUR1**	Sialidase-1		Q99519	0,2
**PGK1**	**PGK1**	Phosphoglycerate kinase 1		P00558	0,2
**HSP90B1**	**ENPL**	Endoplasmin		P14625	0,3
**SERPINB6**	**SPB6**	Serpin B6		P35237	0,3
**PPIC**	**PPIC**	Peptidyl-prolyl cis-trans isomerase C		P45877	0,3
**HSPA13**	**HSP13**	Heat shock 70 kDa protein 13		P48723	0,3
**YWHAQ**	**1433T**	14-3-3 protein theta		P27348	0,3
**MATN2**	**MATN2**	Matrilin-2		O00339	0,3
**PGC**	**PEPC**	Gastricsin		P20142	0,3
**HYOU1**	**HYOU1**	Hypoxia up-regulated protein 1		Q9Y4L1	0,3
**HSPA5**	**GRP78**	78 kDa glucose-regulated protein		P11021	0,3
**LAMC1**	**LAMC1**	Laminin subunit gamma-1		P11047	0,3
**PFN1**	**PROF1**	Profilin-1		P07737	0,3
**SFN**	**1433S**	14-3-3 protein sigma		P31947	0,3
**YWHAB**	**1433B**	14-3-3 protein beta/alpha		P31946	0,3
		Elongation factor 1-alpha (Fragment)		Q53HR5	0,3
**CPZ**	**CBPZ**	Carboxypeptidase Z		Q66K79	0,3
**KLK3**	**KLK3**	Prostate-specific antigen		P07288	0,3
**MUC6**	**MUC6**	Mucin-6		Q6W4X9	0,4
**TUBB2C**	**TBB2C**	Tubulin beta-2C chain		P68371	0,4
**UBC**	**UBC**	Polyubiquitin-C		P0CG48	0,4
**ALB**	**ALBU**	Serum albumin		P02768	0,4
**KRT2**	**K22E**	Keratin, type II cytoskeletal 2 epidermal		P35908	0,4
**GAPDH**	**G3P**	Glyceraldehyde-3-phosphate dehydrogenase		P04406	0,4
**TPI1**	**D3DUS9**	Triosephosphate isomerase		D3DUS9	0,4
**RUVBL1**	**RUVB1**	RuvB-like 1		Q9Y265	0,4
**HSP90AB1**	**HS90B**	Heat shock protein HSP 90-beta		P08238	0,4
**EEF1G**	**EF1G**	Elongation factor 1-gamma		P26641	0,4
**SPA17**	**SP17**	Sperm surface protein Sp17		Q15506	0,4
**LDHC**	**LDHC**	lactate dehydrogenase C chain		P07864	0,4
**PDIA3**	**PDIA3**	Protein disulfide-isomerase A3		P30101	0,4
**HSP90AA1**	**HS90A**	Heat shock protein HSP 90-alpha		P07900	0,4
**SPANXB1**	**SPNXB**	Sperm protein associated with the nucleus on the X chromosome B/F		Q9NS25	0,4
**YWHAE**	**1433E**	14-3-3 protein epsilon		P62258	0,4
**LPL**	**LIPL**	Lipoprotein lipase		P06858	0,4
**KRT9**	**K1C9**	Keratin, type I cytoskeletal 9		P35527	0,4
**ROPN1**	**ROP1A**	Ropporin-1A		Q9HAT0	0,4
**DLAT**	**ODP2**	Dihydrolipoyllysine-residue acetyltransferase of pyruvate dehydrogenase complex		P10515	0,4
**GAPDHS**	**G3PT**	Glyceraldehyde-3-phosphate dehydrogenase, testis-specific		O14556	0,4
**KRT1**	**K2C1**	Keratin, type II cytoskeletal 1		P04264	0,4
**FN1**	**FINC**	Fibronectin		P02751	0,5
**PEBP1**	**PEBP1**	Phosphatidylethanolamine-binding protein 1		P30086	0,5
**YWHAZ**	**1433Z**	14-3-3 protein zeta/delta		P63104	0,5
**ATP5A1**	**ATPA**	ATP synthase subunit alpha, mitochondrial		P25705	0,5
**CCT6A**	**TCPZ**	T-complex protein 1 subunit zeta		P40227	0,5
**KRT10**	**K1C10**	Keratin, type I cytoskeletal 10		P13645	0,5
**PRDX1**	**PRDX1**	Peroxiredoxin-1		Q06830	0,5
**CRISP1**	**CRIS1**	Cysteine-rich secretory protein 1		P54107	0,5
**HSPB1**	**HSPB1**	Heat shock protein beta-1		P04792	0,5
**HSPA2**	**HSP72**	Heat shock-related 70 kDa protein 2		P54652	0,5
**NUCB2**	**D3DQX5**	Nucleobindin 2, isoform CRA_a		D3DQX5	0,5
**GSTM3**	**GSTM3**	Glutathione S-transferase Mu 3		P21266	0,5
**HSPA8**	**HSP7C**	Heat shock cognate 71 kDa protein		P11142	0,5
**ALDOA**	**ALDOA**	Fructose-bisphosphate aldolase A		P04075	0,6
**PRDX5**	**PRDX5**	Peroxiredoxin-5, mitochondrial		P30044	0,6
**OS9**	**OS9**	Protein OS-9		Q13438	0,6
**LAMB2**	**LAMB2**	Laminin subunit beta-2		P55268	0,6
**ATP5B**	**ATPB**	ATP synthase subunit beta, mitochondrial		P06576	0,7
**HSPA1A**	**HSP71**	Heat shock 70 kDa protein 1A/1B		P08107	0,7
**HSPA1L**	**HS71L**	LHeat shock 70 kDa protein 1-like		P34931	0,7
**HSPD1**	**CH60**	60 kDa heat shock protein, mitochondrial		P10809	0,7

Proteins have been listed using the intra and inter-variation analyses-set criteria (CV<0.5 and CV<0.7, respectively).

### Expression Levels of HSPs and PRXs were Consistent Over Time and among Men

Among the 83 consistently expressed proteins (Table 1), we identified chaperones, cytoskeleton proteins, peroxiredoxins, isomerases, and other enzymes. The chaperones identified were: HS90A and HS90B, HSP13, HSP72, HSP7C, ENPL, GRP78, HYOU1, and CLUS. HS90A and HS90B both promote the maturation and structural maintenance of specific target proteins [20], [21]. HSP13 has a peptide-independent ATPase activity [22]. HSP72 is implicated in stabilization of pre-existing proteins [23]. HSP7C acts as a repressor of transcriptional activation [24] and ENPL functions in the processing and transport of secreted proteins [25]. GRP78 is a component of the eIF2 complex, implicated in translational initiation [26]. Finally, HYOU1, a part of a large multi-complex aggregate implicated in cytoprotective mechanisms [27], and CLUS prevent aggregation of non-native proteins [28].

Surprisingly, we found that five of the 14-3-3 family proteins were among the highly-consistent proteins. These are adapter proteins implicated in the regulation of protein function in a large group of signalling pathways [29]. To our knowledge, this is the first report of a number of 14-3-3 family proteins in human spermatozoa.

Four isomerases were found to be highly consistent: D3DUS9 is implicated in glycolysis and energy production [30]; PDIA3 catalyses the formation of disulfide bonds in targeted proteins [31]. PPIB and PPIC are both *cis-trans* isomerases, helping the folding of targeted proteins [32], [33]. Three of the four peroxiredoxins found are associated with the 2-Cys class of peroxiredoxins: PRDX1, PRDX4, and PRDX2. PRDX5 is associated with the atypical 2-Cys class of peroxiredoxins. All of these enzymes are associated with the regulation of oxidation and reduction [34]. Interestingly, we also found several consistently expressed proteins implicated in the regulation of gene expression, such as elongation factor 1-gamma (EF1G) and RUVB1, a component of the NuA4 histone acetyltransferase complex [35].

### Validation of the Variation Analyses by Western Blotting

**Figure 5:**
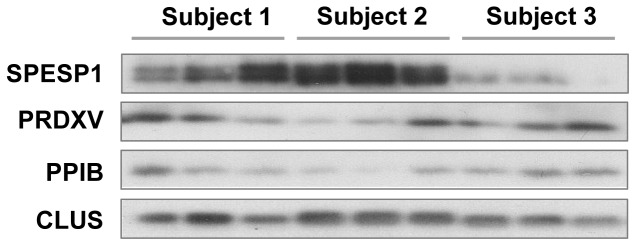
Validation of the intra and inter-individual analysis by western blotting. The coefficient of variation of four proteins with various dispersion levels was calculated by western blotting. The intra and inter-individual variation analysis based on proteomic quantitative data (n = 3) were compared with that obtained by western blot analysis of the three subjects used for the proteomic analysis and three additional subjects. The table 2 regroups the average of the intra CV and the inter CV calculated from the proteomic results (n = 3) and the CV calculated from the western blot data (n = 6).

In order to confirm the dispersion level found by the inter-individual analyses of the proteomic data, the coefficients of variation of four proteins with various dispersion levels were analysed by western blotting (Fig. 5 and Fig. S2). The intra-individual variation was evaluated from the data of the three subjects used for the proteomic analysis and the three subject added for the Western blot experiment (n = 6).The average of the intra-individual CV and the inter-individual CV are shown in table 2. The average of the intra-individual CV based on the western blot data showed consistency with the CV calculated using the proteomic quantitative data. The inter-individual CV, based on western blot data, show that the variation is less than that described with the proteomic quantitative data. Nevertheless, a protein described as stable (PPIB) or variable (SPESP1) by proteomic analysis appears to be similarly changed in western blot analysis. These results show that after the application of all criteria, calculation of the coefficient of variation from proteomic data is valid.

**Table 2:** Average of the intra and inter coefficient of variation (CV).

	Average Intra CV		Inter CV	
	Proteomic n = 3	Western Blot n = 6	Proteomic n = 3	Western Blot n = 6
**SPESP1**	0.3	0.3	0.8	0.6
**PRDXV**	0.3	0.3	0.6	0.4
**PPIB**	0.1	0.1	0.3	0.2
**CLUS**	0.1	0.1	0.1	0.1

The intra and inter CV were calculated from the proteomic results (n = 3) and the CV calculated from the western blot data (n = 6).

## Discussion

The methodology used in proteomics is particularly suitable to the study of spermatozoa because these cells are transcriptionally silent, i.e., the nature of the protein profile reflects the functional status of sperm. While proteomic analysis allows us to generate inventories of thousands of proteins expressed in sperm [36], comparative studies are useful in finding a link between the listed proteins and their biological role. Differences in the identity of proteins and their quantitative levels of expression may be a consequence of basic variations in the biological process of spermatogenesis and/or maturation in the epididymis or due to genetic polymorphisms.

To our knowledge, this is the first study describing quantitative expression variations in the sperm head enriched proteome in fertile men. Through the quantitative measurement of sperm head proteins, we were able to compare their expression levels across different times of collection for individual subjects and across different subjects. An important strength of our study was the use of technical triplicate analyses for each individual at each time point, allowing us to evaluate and exclude contextual background protein identifications associated with technical variations inherent to the methodology and analyses. Application of identification and quantification cut-off criteria decreased the maximum variance of the mean of the three estimated quantitative values by nearly 3-fold. This observation shows the degree of arbitrary conclusions that may be derived if no replicate is used and demonstrates the importance of defining a threshold of inclusion of protein signals before conducting comparative analyses.

Another important feature of our study design was the variation analyses of samples collected at different time points using semen samples from each subject. This approach allowed us to identify proteins that are consistently found both over time within an individual and among a cohort of men with a defined reproductive phenotype (normozoospermia). The identification of these proteins, for which there is a high level of confidence, serves as the basis for further analyses in defining the sperm head protein profile of a subject. Judging from their consistent presence among all subjects evaluated, they are likely to also play essential roles in normal sperm function. Our approach also allowed us to identify a list of proteins that were highly variable across time and not shared among subjects. Our findings highlight the importance of using multiple subjects and time points in the comparative proteomic analysis of sperm.

Albumin (ALBU) is known to be the major protein detected from seminal plasma. In our study, ALBU was detected at a very low level, with an average of 0.05% of total spectra, indicating that seminal protein contamination is very low. Proteins that were expressed only at a low level were excluded by application of the cut-off before the quantitative analysis.

Although the spectral counting quantitation approach is simpler to do, its reliability is restricted to a limited dynamic range of concentrations, particularly when dealing with proteins for which only a handful of spectra are identified. The ion profiling quantitative approach is used both as a means to ameliorate the relative quantitative determination of proteins at both extremes of the spectral abundance for which subtle differences in expression may have gone undetected by the spectral counting approach and as a way to validate those determinations for which both methods are in agreement. In our study, we used determinations from these two separate quantification methods to compare the dispersion of the expression levels of proteins found to constitute the core proteome across time and among subjects. Comparison of the CVs revealed that no significant differences were found in the distribution of the CVs obtained by both methods. This indicates that for relatively small fold differences both methods are fairly reliable; furthermore, the range of expression levels does significantly affect the reliability of the determination of ratios.

The magnitude of the dynamic range of proteins in a complete spermatozoon is evaluated at 10^5–6^ [37]. Our preparations of sperm were specifically enriched in sperm head proteins to reduce the levels of complexity (i.e., eliminating most tail sperm proteins from the biological samples studied), allowing us to identify a total of 686 proteins. Among these, 22% (n = 149) were identified as nuclear proteins based on information derived from somatic cells. When comparing total spectra abundance in one sample, the relative ratios of known spermatozoon nuclear proteins, such as H4 and H2A.J, were found to be 1.0 and 2.5 fold higher than the level of AKAP4, the most abundant protein known to be expressed in the entire spermatozoon [37]. This reflects the success of our methodology in enriching sperm heads. The true proportion of nuclear proteins in the spermatozoon, however, cannot be accurately defined since not all nuclear proteins in spermatozoa have been identified and localized.

A total of 83 proteins, representing 75% of the total proteins included in the inter-individual analysis of variation (Table 1), were commonly found in the core proteome across time and among subjects. Several HSPs were expressed in sperm and some of these were expressed at high levels [37]; HSPs have been associated with capacitation and indirectly with sperm-egg binding [37]. Their specific functions seem to depend on their tyrosine phosphorylation state and whether they migrate to the surface of the spermatozoon [37]. In our study, we also found that the expression of nine chaperones was highly consistent. A recent study showed that three of these chaperones, namely, HSP7C, GRP78 and HYOU1, are expressed in the plasma membranes of human spermatozoa and are accessible to surface labelling [38].

Another protein that was found to be highly consistently expressed was the T-complex protein 1 subunit zeta (TCPZ). Interestingly, TCPZ has been described as a component of chaperonin-containing TCP-1 complex [39]. This multimeric protein complex is involved in sperm-zona pellucida interaction and TCPZ protein co-localizes with the ZPBP2 protein [39].

Capacitation is required to ensure the fertilization ability of spermatozoa. This complex process is highly regulated by phosphorylation of proteins and controlled by the amount of reactive oxygen species (ROS) [40]. Spermatozoa themselves are capable of producing ROS that, at low levels, are required for capacitation and hyperactivation [41]. However, high levels of ROS could result in oxidative stress and DNA damage. In fact, high ROS levels are detected in 25% of infertile men [42], [43]. Peroxiredoxins are important in redox regulation in somatic cells [34]. In our study, we found four peroxiredoxins, namely, PRDX1, PRDX2, PRDX4 and PRDX5, to be expressed consistently across time and among men. PRDX proteins are found in multiple compartments in the human spermatozoon; PRDX4 is localized in the acrosome, while PRDX1 is in the equatorial segment and PRDX5 is in the post-acrosomal region [44]. Gong et al., 2012 [45] noted the relationship of PRDX1 with fertility status.

Semenogelins prevent capacitation by reducing ROS production and hyperactivated motility; they are the main component of the human semen coagulum [46], [47]. Recently, ROS in spermatozoa have been shown to have an impact on semenogelin metabolism [48], indicating a co-regulation of ROS and semenogelins, both involved in delaying the initiation of capacitation.

This study represents the first description of the analyses of intra- and inter-individual variation of the sperm head proteome based on quantitative observations. We have clearly demonstrated the necessity of using more than one sample per subject in comparative studies; triplicate analysis of each sample helped to minimize haphazard quantification of the proteins with a very low level of expression. This study serves to increase our knowledge about proteins expressed in sperm. Furthermore, it lays the foundation for future comparative studies aimed at correlating protein profile expression with various pathological conditions, such as male infertility.

## Supporting Information

Figure S1 **Gene ontology annotation of the 686 proteins identified.** Pie chart based on gene ontology tool from Scaffold software illustrating the distribution of proteins in the molecular function and cellular component categories.The majority of identified proteins are associated with molecular functions (n = 542), such as isomerases, and binding (n = 455) in the case of chaperones. Some proteins are associated with transport activity (n = 33), such as importins, and others with antioxidant activity (n = 12), such as peroxiredoxins. Interestingly, some of the proteins are associated with transcription regulation activity (n = 15), e.g., ribosomal proteins. Based on cellular components, the majority of proteins were associated with cytoplasm (n = 404), intracellular organelles, (n = 394), organelle parts (n = 261), membranes (n = 186), extracellular regions (n = 142), or the nucleus (n = 149).
(TIF)

Figure S2 **Western blot data of the proteins from the sperm sample of the three added control men.** Proteins extracted from the sperm head-enriched samples from the three subjects of the study were resolved by SDS page and immunoblotted with antibodies. The coefficients of variation of those proteins were compared with the ones based on proteomic quantitative data.
(TIF)

File S1 **Contains: Table S1. Semen parameters data from the six subjects.** The average age of the three subjects included in the proteomic analysis was 35.7 yrs (30, 38, and 39 years). Two of the subjects had a previous history of natural fecundity. The third one had not previously attempted to achieve pregnancy with partners. One subject had a history of hypertension that was well-controlled by medication with no additional significant co-morbidities. Another subject had a history of tobacco consumption that had been terminated for more than three years. Neither of the other subjects reported a history of smoking or illicit drug use**.** The average age of the three subjects added for the western blot analysis, were 24 (22–26) yrs. None of these three subjects have any significant co-morbidity. There was no history of smoking or use of any drugs and medications. All six subjects had values that placed them in the fertile range, as per WHO standards. **Table S2.** **List of the 686 identified proteins by LC-MS/MS analysis**. Each identified protein is listed from the most abundant to the less abundant. **Table S3**. **List of protein analysed in the intra-individual and inter-individual analyses**. Each protein included in the variation analyses for each subject is listed depending its variation level. The lowest is the CV, the lowest is the variation level over-time and/or among subject.
(XLSX)
