# Supplementary figures and images for: Analysis of the Sperm Head Protein Profiles in Fertile Men: Consistency across Time in the Levels of Expression of Heat Shock Proteins and Peroxiredoxins
Source: PLoS One. 2013 Oct 30;8(10):e77471. doi: 10.1371/journal.pone.0077471 (PMC3813703; doi:10.1371/journal.pone.0077471)

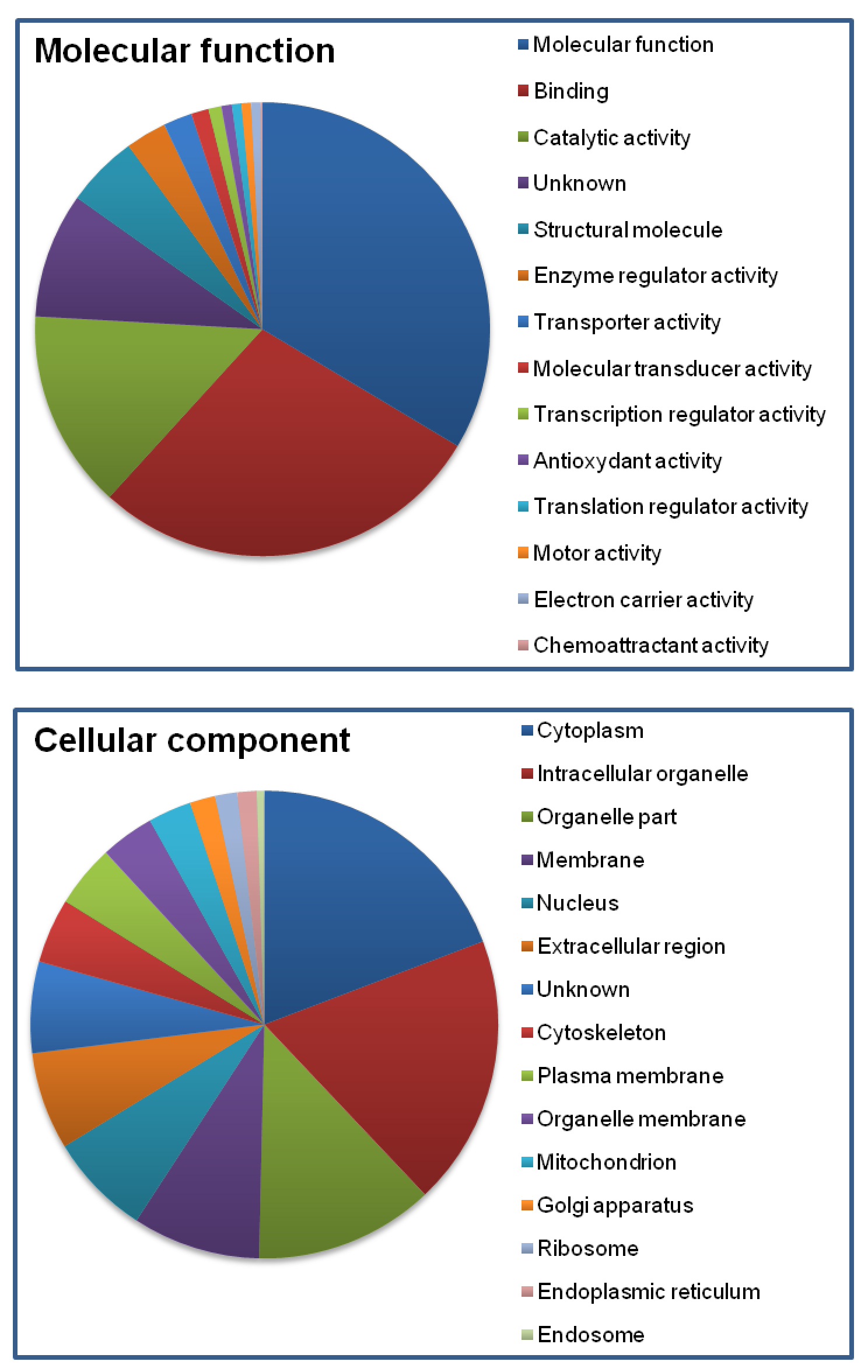

Supplement: Figure S1 — Gene ontology annotation of the 686 proteins identified. Pie chart based on gene ontology tool from Scaffold software illustrating the distribution of proteins in the molecular function and cellular component categories.The majority of identified proteins are associated with molecular functions (n = 542), such as isomerases, and binding (n = 455) in the case of chaperones. Some proteins are associated with transport activity (n = 33), such as importins, and others with antioxidant activity (n = 12), such as peroxiredoxins. Interestingly, some of the proteins are associated with transcription regulation activity (n = 15), e.g., ribosomal proteins. Based on cellular components, the majority of proteins were associated with cytoplasm (n = 404), intracellular organelles, (n = 394), organelle parts (n = 261), membranes (n = 186), extracellular regions (n = 142), or the nucleus (n = 149). (TIF) [file pone.0077471.s001.tif]

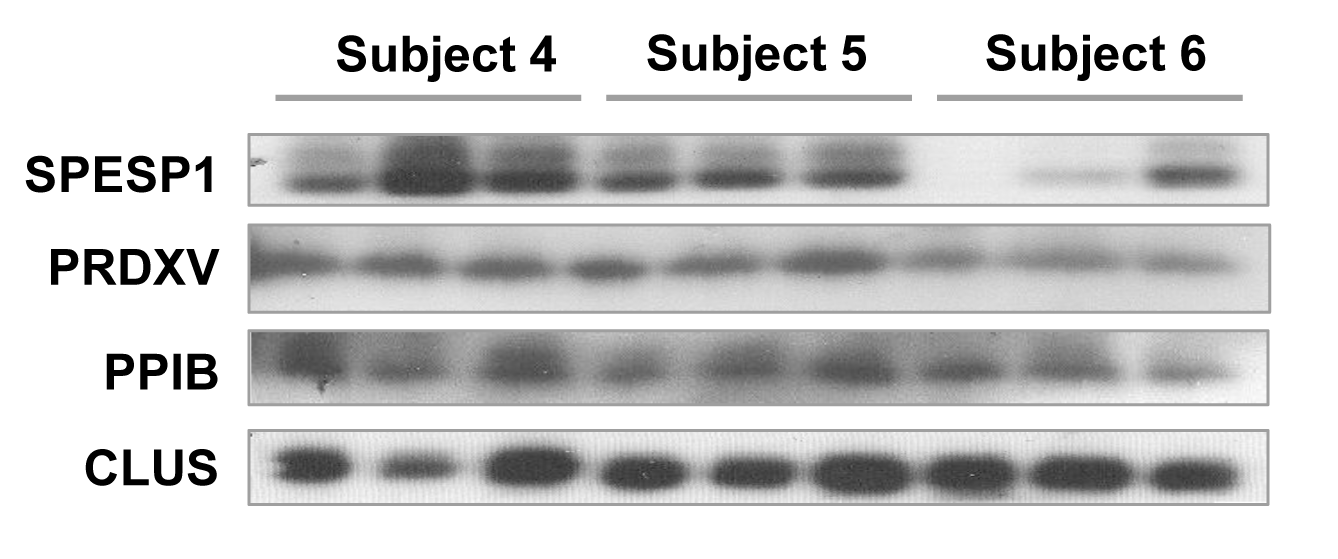

Supplement: Figure S2 — Western blot data of the proteins from the sperm sample of the three added control men. Proteins extracted from the sperm head-enriched samples from the three subjects of the study were resolved by SDS page and immunoblotted with antibodies. The coefficients of variation of those proteins were compared with the ones based on proteomic quantitative data. (TIF) [file pone.0077471.s002.tif]
